# A novel fluorescent reporter sensitive to serine mis-incorporation

**DOI:** 10.1080/15476286.2021.2015173

**Published:** 2022-02-15

**Authors:** Peter Rozik, Robert Szabla, Jeremy T. Lant, Rashmi Kiri, David E. Wright, Murray Junop, Patrick O’Donoghue

**Affiliations:** a Department of Biochemistry, The University of Western Ontario, London, Ontario, Canada; b Department of Chemistry, The University of Western Ontario, London, Ontario, Canada

**Keywords:** Fluorescent live-cell reporter, genetic code, mistranslation, protein synthesis, transfer RNA, translation fidelity

## Abstract

High-fidelity translation was considered a requirement for living cells. The frozen accident theory suggested that any deviation from the standard genetic code should result in the production of so much mis-made and non-functional proteins that cells cannot remain viable. Studies in bacterial, yeast, and mammalian cells show that significant levels of mistranslation (1–10% per codon) can be tolerated or even beneficial under conditions of oxidative stress. Single tRNA mutants, which occur naturally in the human population, can lead to amino acid mis-incorporation at a codon or set of codons. The rate or level of mistranslation can be difficult or impossible to measure in live cells. We developed a novel red fluorescent protein reporter that is sensitive to serine (Ser) mis-incorporation at proline (Pro) codons. The mCherry Ser151Pro mutant is efficiently produced in *Escherichia coli* but non-fluorescent. We demonstrated in cells and with purified mCherry protein that the fluorescence of mCherry Ser151Pro is rescued by two different tRNA^Ser^ gene variants that were mutated to contain the Pro (UGG) anticodon. Ser mis-incorporation was confirmed by mass spectrometry. Remarkably, *E. coli* tolerated mistranslation rates of ~10% per codon with negligible reduction in growth rate. Conformational sampling simulations revealed that the Ser151Pro mutant leads to significant changes in the conformational freedom of the chromophore precursor, which is indicative of a defect in chromophore maturation. Together our data suggest that the mCherry Ser151 mutants may be used to report Ser mis-incorporation at multiple other codons, further expanding the ability to measure mistranslation in living cells.

## Introduction

Mistranslation is the phenomenon of errors in protein synthesis due to mutations in tRNAs [1], aminoacyl-tRNA synthetases, ribosomes, or other components of the protein quality control machinery [2,3]. Mutations either in the recognition elements of tRNAs or their anticodons can lead to incorporation of the wrong amino acid in response to a codon or set of codons, affecting all proteins. The frozen accident theory suggested that any deviation from the standard genetic code results in so many mis-made and non-functional proteins that cells cannot remain viable [4]. The ability of tRNAs to mis-incorporate amino acids [5] was first observed two years before the table of codon to amino acid assignments was completely known [6,7]. The study revealed a glycine residue mutated in arginine led to inactive tryptophan synthetase and tryptophan auxotrophy in *Escherichia coli*. Several suppressor mutants were uncovered that produced functional tryptophan synthetase by mis-incorporating glycine at the mutant arginine codon. Mis-incorporation represented a minority of the protein produced that was sufficient to enable viability without tryptophan. The suppressor was supposed and later confirmed to be a tRNA^Gly^ that mistranslates the arginine ^5ʹ-^AGA^−3ʹ^ codon with glycine [8,9] as a result of an anticodon change from Gly (^5ʹ-^UCC^−3ʹ^) to Arg (^5ʹ-^UCU^−3ʹ^) [10].

In the information transfer from DNA to RNA to protein, translation of mRNA is the most error prone step [11]. Protein abundance and error levels are dependent on the contributions of several factors, including aminoacyl-tRNA de-coding efficiency on the ribosome, transcript abundance, translation rates, and the rate of protein and mRNA degradation. Mistranslation or error rates in protein synthesis are normally described in terms of the fraction of errors that occur per codon. Indeed, translation error rates per codon are considered low as estimates suggest that normal cells mis-read 1 in every 1,000 to 10,000 codons [12,13]. Fascinatingly, significantly higher levels of mistranslation (1–10% per codon) can be tolerated in bacterial [14], yeast [15] and mammalian cells [16]. Mistranslation can even be beneficial in conditions of stress [17], including oxidative stress as suggested by studies of methionine mis-incorporation in human cells [18]. In *E. coli*, moderate levels of mis-incorporation protect cells against oxidative damage by inducing the expression of anti-oxidant genes [19], while studies in yeast found mis-incorporation led to oxidative damage to lipids [20]. These findings suggest that diverse cells can tolerate higher levels of mistranslation than initially assumed.

Single tRNA mutants can lead to amino acid mis-incorporation in human cells [21,22]. Indeed, human tRNA variants that cause loss of function are already implicated in disease [14]. Mistranslation can also lead to or exacerbate diseases, including neurological disorders [22,23]. A missense mutation in the alanyl-tRNA synthetase gene causes cerebral cell loss of terminally differentiated neurons in mice [23]. The accumulation of misfolded protein inevitably led to cell death, a seminal discovery that provided a novel mechanism connecting mistranslation to neurodegeneration. Other alanyl-tRNA synthetase mutants cause neurodegenerative diseases, including Charcot-Marie-Tooth disease [24] and early-onset epileptic encephalopathy [25]. While we anticipate that higher rates of mistranslation will lead to increased cell stress and toxicity, for most types of mistranslation there are no adequate reporters to quantify mis-incorporation in live cells.

Here, we report on a serine-to-proline mutation at position 151 in mCherry that eliminates fluorescence. Because the seryl-tRNA synthetase (SerRS) does not recognize any of the anticodon bases [26], mutations to the tRNA^Ser^ anticodon will lead to serine mis-incorporation. We found that fluorescence of the mCherry Ser151Pro mutant was effectively rescued only in cells expressing tRNA^Ser^ variants with Pro (UGG) anticodons. Thus, mistranslating cells were able to produce a fraction of wild-type mCherry from the mutant allele. We used mass spectrometry to confirm serine mis-incorporation in mCherry. Conformational sampling simulations of the mCherry variants showed that the conformational freedom of the chromophore precursor is significantly altered in the Ser151Pro mutant. Together our data provide a mechanistic basis for the elimination and rescue of fluorescence in the mCherry Ser151Pro variant in response to mistranslation in living cells.

## Methods

### Plasmids, strains, and cloning

The pET28a-His-TEV-mCherry (kanamycin resistant) plasmid was a kind gift of Dr. Donald E. Spratt [27]. The pGFIB [28] (ampicillin resistant) constructs were cloned containing expression cassettes for two different tRNA^Ser^ genes and two corresponding mutants with the UGG (Pro) anticodon (tRNA^Ser^_UGG_). The *E. coli* tRNA^Ser^ GGA-1-1 gene has a GGA anticodon (tRNA^Ser^_GGA_) and the other tRNA^Ser^ gene we investigated here (GCT-1-1) has a GCU anticodon (tRNA^Ser^_GCU_). Plasmid manipulations were done in *E. coli* DH5α. The wild-type and UGG anticodon variants of *E. coli* tRNA genes GGA-1-1 and GCT-1-1 were synthesized as complementary DNA oligomers (Sigma-Aldrich, Oakville, ON) with 5ʹ-*Eco*RI and 3ʹ-*Bam*HI restriction sites. Following hybridization of forward and reverse oligos, the product was ligated with T4 DNA ligase (New England Biolabs, Ipswich, MA, USA) into an *Eco*RI/*Bam*HI digested and gel-purified pGFIB vector. Successful clones were verified by DNA sequencing at the London Regional Genomics Centre (Western University, London, Ontario).

### Transformation and analysis of mCherry libraries

Mutagenic primers (Table S1) with degenerate NNS codons targeting Ala49, Ser74, and Ser151 of mCherry were ordered from Sigma-Aldrich (Oakville, ON, Canada) and then phosphorylated using T4 polynucleotide kinase (NEB). Each of the three targeted residues in mCherry (pET28a-HisTev-mCherry [27]) was separately mutagenized by round-the-horn PCR site-directed mutagenesis [29] and ligated with T4 DNA ligase (NEB). The ligated products were transformed into *E. coli* BL21(DE3) cells (EdgeBio, Gaithersburg, MD, USA) with super optimal broth with catabolite repression (SOC) as recovery media and plated on Lysogeny Broth (LB) agar supplemented with kanamycin (25 μg/mL). Colonies formed over 24-hour incubation at 37°C and were then imaged with bright field and fluorescence (ex 570 nm, em 610 nm) in a Bio-Rad Chemidoc MP. For selected colonies, liquid cultures (5 mL) were grown in LB with kanamycin overnight at 37 °C and plasmid DNA was purified (GeneAid, New Taipei City, Taiwan). Successful cloning of the Ser151Pro mutant, characterized here, as well as Ser151Phe was verified by DNA sequencing at Genewiz (Cambridge, MA, USA).

### Growth analysis and fluorescence quantitation

The plasmids pGFIB expressing wild-type or mutant tRNAs, and pET28a expressing wild-type mCherry or Ser151Pro were co-transformed into competent *E. coli* BL21 cells (Invitrogen, Carlsbad, CA, USA). Individual transformant colonies from selective plates (LB agar with 100 μg/mL ampicillin, 25 μg/mL kanamycin) representing three biological replicates were used to inoculate 10 mL LB pre-cultures, which were grown overnight, shaking at 37°C. The grown pre-cultures were aliquoted and diluted with additional media to a standardized A_600_ = 1.0. We assumed that one A_600_ unit = 8 × 10^8^ cells/mL. From the standardized cultures, a 1 μL volume of cells was aliquoted onto LB agar plates with antibiotics and 0.1 mM isopropyl β-D-1-thiogalactopyranoside (IPTG) to induce mCherry expression. Colonies formed during a 24-hour incubation at 37°C and were then imaged with bright field and fluorescence (ex 570 nm, em 610 nm) in a Bio-Rad Chemidoc MP. The grown pre-cultures were also diluted in LB to a standardized A_600_ = 0.1. From the diluted cultures, 200 μL was added to each well of a 96-well microplate representing three technical replicates for each of three biological replicates. Subsequently, IPTG was added to each well to a final concentration of 0.1 mM to induce mCherry expression. Using a BioTek Synergy H1 microplate reader, the cells were incubated at 37°C while shaking and monitored over a 16 hr time course. A_600_ and fluorescence (ex 570 nm, em 610 nm) were recorded in 15 min intervals.

### Affinity purification of mCherry variants

The mCherry proteins were isolated using His-tag affinity purification. Starting from 10 mL pre-cultures, BL21 strains expressing mCherry and tRNA variants were grown at 37°C in 1 L of LB with vigorous shaking and antibiotics (ampicillin 100 μg/mL; kanamycin 25 μg/mL). At A_600_ = 0.6–0.7, IPTG (0.1 mM) was added to induce mCherry production and the temperature was lowered to 20°C. Following 14–16 hrs of induction, cells were then harvested and lysed via sonication on ice (70% Amplitude, 1 sec pulse, 1 sec pause) in buffer A (50 mM NaH_2_PO_4_, 300 mM NaCl, 10 mM imidazole, 10% glycerol, pH 7.2) and lysozyme was added to a concentration of 300 μg/mL along with 1 tablet of EDTA-free mini protease inhibitor mixture (Roche Applied Science). Centrifugation at 15,000 × g for 1 hr separated the soluble lysate supernatant from the insoluble pellet. The lysate was filtered using a 2 μm sterile filter. The filtered lysate was loaded onto a Ni^2+^-nitrilotriacetic acid (NTA) column that had been pre-equilibrated with 10 column volumes of buffer containing 50 mM NaH_2_PO_4_, 300 mM NaCl, 20 mM imidazole, 10% glycerol, pH 7.2. The column was washed with 10 column volumes of wash buffer (50 mM NaH_2_PO_4_, 300 mM NaCl, 10% glycerol, pH 7.2 containing 40 mM imidazole) followed by 10 column volumes of the same buffer containing 50 mM of imidazole. The protein was eluted in the same buffer containing 300 mM imidazole. The protein was dialysed overnight into storage buffer (50 mM NaH_2_PO_4_, 300 mM NaCl, 50% glycerol, pH 7.2) and stored at −80°C for further analysis.

### Fluorescent quantitation of purified protein

Protein concentrations were determined using a standard Bradford assay. Purified mCherry protein variants were aliquoted in a serial dilution (20 µg to 0.625 µg) in buffer A lacking imidazole on a microplate. The aliquoted protein samples, representing three technical replicates for each of the three biological replicates, were then placed in a BioTek Synergy H1 microplate reader to measure mCherry fluorescence (ex 570 nm, em 610 nm) in each sample.

### Tandem mass spectrometry (MS/MS) analysis

The mCherry proteins were separated using 12% sodium dodecyl sulphate polyacrylamide gel electrophoresis (SDS-PAGE) and visualized following staining with Coomassie blue. The bands were excised and digested using an automated in-gel digestion at the Functional Proteomics Facility (London, Ontario). Proteolytic digestions were performed with trypsin. The tryptic digested peptides were then subjected to reversed phase ultra-pressure liquid chromatography using a Waters nanoAcquity system coupled to a Fourier transform (FT) Thermo Scientific Orbitrap Elite mass spectrometer using a Nanoflex source. The samples were trapped and then eluted onto a Waters BEH C18 75 μm × 25 cm column using a gradient of 5–40% buffer B (acetonitrile + 0.1% formic acid) over 90 min. The MS mass range was set at 400–1800 *m*/*z* with a high-resolution set at 120,000 and the MS^2^ scans were set using a fixed first mass of 100 Da using a FT/FT/HCD data-dependent acquisition scheme. Higher-energy C-trap dissociation (HCD) scans were detected in the orbitrap with a three-step collision energy of 38 ± 20%. Raw data were visualized with the Qual Browser feature of the Thermo Scientific XCalibur software, and data analysis was performed using PEAKS 7.0 (Bioinformatic Solutions Inc, Waterloo, Ontario) [30]. Peptide sequences were generated by *de novo* sequencing. The data were also searched against the Swiss-Prot protein sequence database to match unmodified peptides as well as a custom database containing all possible Pro to Ser substitutions in the mCherry sequence. Additionally, the spectra were searched for all possible single amino acid substitutions.

### Conformational sampling of the mCherry chromophore precursor

All mCherry modelling was initiated from the highest-resolution mCherry crystal structure available in the PDB (PDB accession 2H5Q at 1.36 Å) [31]. First, the mature chromophore CH6 was removed and remodelled in the form of its precursor peptide, Met71-Tyr72-Gly73 using the Rosetta Kinematic Loop Closure with Fragments (KIC) protocol [32]. The precursor peptide was remodelled along with flanking residues that comprise the central helix of mCherry: Phe56 to Lys79. Rosetta KIC was set up to generate 1,000 mCherry precursor models. The lowest-energy precursor model was identified and used for all downstream conformational sampling. Global conformation sampling of the entire precursor protein conducted on wild-type, Ala150Gly, Ser151Pro and Ser151Phe variants of mCherry using the Rosetta Relax protocol [33]. A set of 1,000 potential mCherry conformers was generated for each mutant. All Cα atoms in each conformer were aligned to the reference PDB structure 2H5Q [31]. While in this aligned state, root mean square deviation (RMSD) of the Met71-Tyr72-Gly73 tripeptide was calculated against the input model using a custom PyMOL script. The distribution in RMSD values was compared between all pairs of mCherry mutants using the non-parametric two-sample Kolmogorov–Smirnov test [34]. This was done using the ks_2samp module in the SciPY python package [35]. All Rosetta computations were executed on the Compute Canada Niagara HPC cluster running Rosetta 3.10. The Rosetta commands used are provided in the supplemental material.

## Results

### Identifying fluorescence-disabling mCherry mutants

To generate new mistranslation-sensitive reporters based on mCherry, we selected three sites to randomly mutagenize. Two residues (Ser74 and Ser151) were selected as potential reporters for serine mis-incorporation, while we selected Ala49 as a possible reporter for alanine mis-incorporation. We transformed PCR-based mutagenic libraries with the NNS codon replacing either Ala49, Ser74, or Ser151. Transformation of each library onto selective agar plates led to colony growth observed the following day. A fraction (~11%) of the colonies in the Ser74X library retained robust fluorescence (Figure S1A), suggesting this site may be permissive to multiple amino acid replacements and perhaps not suitable as a mis-incorporation reporter. With only one exception, all the colonies from the Ser151X (Figure S1B) and Ala49X (Figure S1C) library were non-fluorescent.

While further characterization of the mutant libraries is beyond the scope of the current work, we sequenced selected plasmids derived from colonies from the Ser151X libraries to determine the nature of the mutation and fluorescent properties of the mCherry mutants. We then independently re-cloned and characterized two Ser151X mutants further. We also compared growth and fluorescence to a wild-type mCherry and a Ala150Gly mutant that we made in an attempt to create an alanine mis-incorporation reporter. On solid media, we found that *E. coli* cells expressing the wild-type, Ala150Gly, Ser151Pro, and Ser151Phe variants all showed similar growth (Figure S2). Fluorescent images document that the wild-type and Ala150Gly variants showed robust mCherry fluorescence (Figure S2A). We produced the wild-type mCherry and variants Ser151Pro and Ser151Phe in *E.* *coli* BL21 and analysed the resulting lysates using SDS-PAGE, Coomassie staining, and Western blotting. An immunoblot for the His-tag (Figure S2B) showed that despite the lack of fluorescence in the Ser151Pro and Phe mutants, both proteins were produced at a similar level to the wild-type mCherry in *E. coli*. The data suggest that Ser151 may serve as a site to report serine mis-incorporation at multiple codons.

### mCherry Ser151Pro can be rescued by tRNA-dependent mistranslation

**Figure 1.**
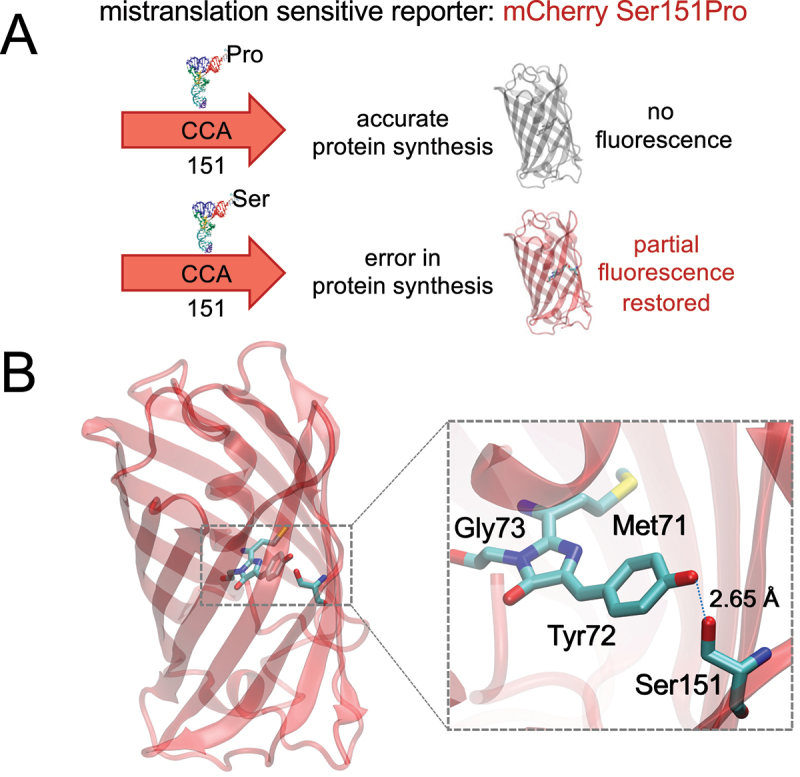
Design of a mistranslation sensitive fluorescent protein. To detect mistranslation events, a mutant tRNA^Ser^ with a proline anticodon is co-expressed with a fluorescently inactive mCherry Ser151Pro mutant (a). If no mistranslation event occurs at position 151, then the mCherry is accurately made and does not fluoresce. A mistranslation event at position 151 that incorporates serine instead of proline will restore fluorescent activity. Ser151 interacts with the mCherry chromophore (Met71-Tyr72-Gly73, PDB code 2H5Q [31]) via a hydrogen bond (b). We anticipated that mutation at Ser151 would interfere with the chromophore and disrupt mCherry fluorescence.

**Figure 2.**
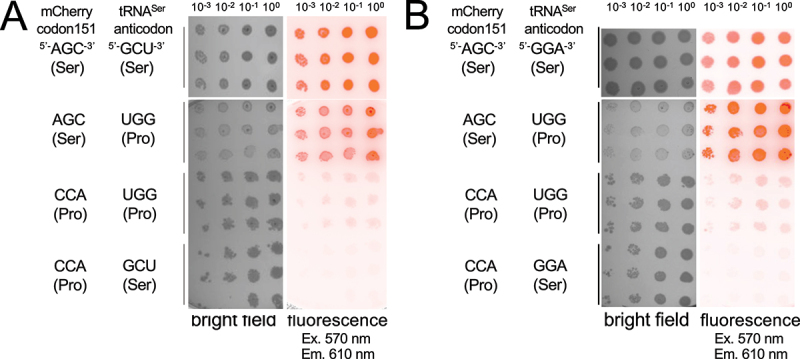
Growth on solid media of wild-type or mistranslating *E. coli* expressing mCherry variants. *E. coli* BL21 cells were transformed with plasmids expressing either wild-type mCherry with a Ser151 AGC codon or mutant mCherry with a Pro151 CCA codon. Each strain was co-transformed with a second plasmid bearing either wild-type tRNA^Ser^, derived from the GCT 1–1 tRNA^Ser^ gene (a) or the GGA 1–1 tRNA^Ser^ gene (b), or a mutant version of each tRNA^Ser^ gene that has a UGG proline-decoding anticodon. mCherry with a Pro151 codon co-expressed with either of the tRNA^Ser^_UGG_ variants demonstrated fluorescence rescue resulting from mistranslation. For the negative controls, a wild-type tRNA^Ser^ was co-expressed with a mutant mCherry Pro151 that did not fluoresce above the background. Cultures from three biological replicates were aliquoted in serial dilution as indicated in Methods.

We hypothesized that co-expression of the mutant tRNA^Ser^_UGG_ would cause mis-incorporation of Ser at Pro codons at a rate that is proportional to the fluorescence recovered from the mCherry Ser151Pro mutant. We employed two different tRNA^Ser^ mutants that both include the proline-decoding UGG anticodon. Our analysis of the mCherry structure indicated that Ser151Pro may perturb the chromophore confirmation (Figure 1(b)). As noted above, Ser151Pro does not fluoresce (Figure S2). Cells expressing either the wild-type or Ser151Pro mutant mCherry along with either a wild-type tRNA^Ser^ (tRNA^Ser^_GGA_ or tRNA^Ser^_GCU_) or the corresponding mutant tRNA^Ser^_UGG_ were grown in liquid pre-cultures and then aliquoted in serial dilutions on solid selective media (Figure 2). We observed similar growth in all cell lines with no stark differences in growth between wild-type and mistranslating cells. Fluorescent imaging showed robust fluorescence of the wild-type mCherry when it was expressed in either normal or mistranslating cells. No above-background fluorescence of mCherry Ser151Pro was detected in normal cells; however, fluorescence recovery was evident in mistranslating cells co-expressing mCherry Ser151Pro (Figure 2). In this assay, we observed a similar level of fluorescent recovery from either of the tRNA^Ser^_GCU_ (Figure 2(a)) or the tRNA^Ser^_GGA_ (Figure 2(b)) derived proline-decoding UGG anticodon mutants. The mCherry reporter, thus, enables visualization of mistranslation events in live cells (Figure 2).

**Figure 3.**
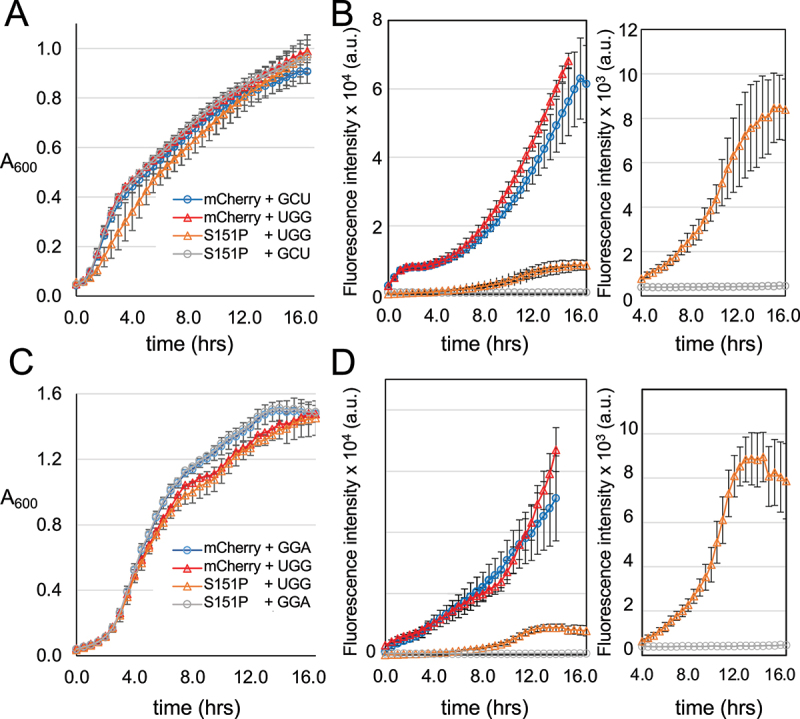
Growth of *E. coli* strains expressing wild-type or mistranslating tRNAs. Using a 96-well microplate, cell growth (A_600_) was recorded over a 16-hour time course for (a) *E. coli* cells co-expressing mCherry with tRNA^Ser^_GCU_ (blue circles), mCherry with mutant tRNA^Ser^_UGG_ (red triangles), mCherry Ser151Pro with tRNA^Ser^_UGG_ (Orange triangles), or mCherry Ser151Pro with wild-type tRNA^Ser^_GCU_ (grey circles). Cell growth (c) is also shown for *E. coli* cells expressing mCherry with tRNA^Ser^_GGA_ (blue circles), mCherry with a corresponding mutant tRNA^Ser^_UGG_ (red triangles), Ser151Pro with mutant tRNA^Ser^_UGG_ (Orange triangles), Ser151Pro with tRNA^Ser^_GGA_ (grey circles). At each time point, fluorescence (ex 570 nm, em 610 nm) intensity was also recorded (b, d). The inset panels (b, d) show a zoomed in view of the fluorescence recovery due to serine mis-incorporation at Pro151.

**Figure 4.**
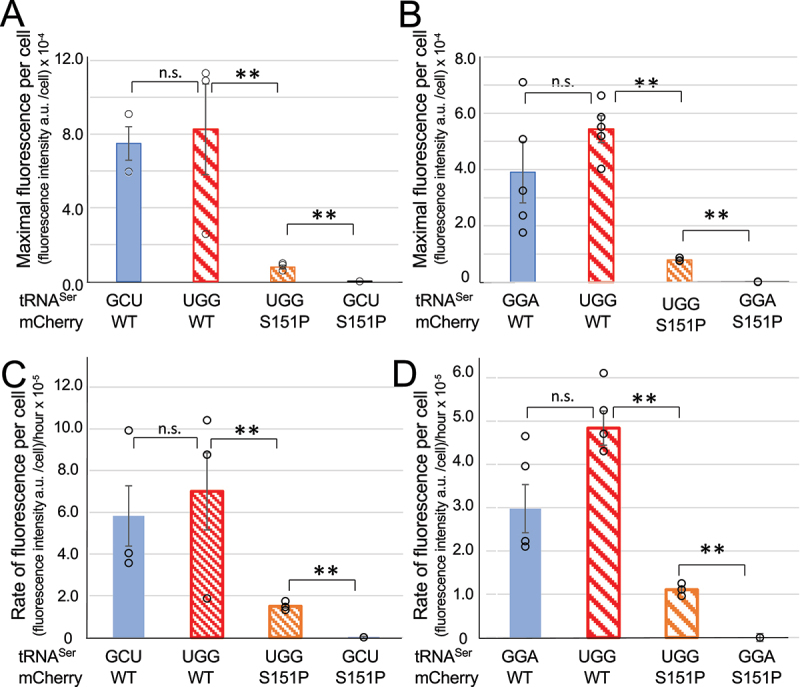
Quantification of mistranslation level and rate in live *E. coli* cells. Data from cell growth and mCherry fluorescence in microplates (Figure 3) were analysed to compare the maximal fluorescence per cell observed in each of the indicated mCherry producing strain (a, b). The rate of fluorescence increase per cell (c, d) was calculated by measuring the slope of the linear phase of fluorescent mCherry production per cell (Figure 3(b,d)). Data are shown for the tRNA^Ser^_GCU_ derived variants (a, c) and for the tRNA^Ser^_GGA_ derived variants (b, d). Error bars represent ± 1 standard deviation of three biological replicates and three technical replicates. Statistical significance is based on ANOVA single factor analysis (n. s. – not significant, ** p < 0.01).

**Figure 5.**
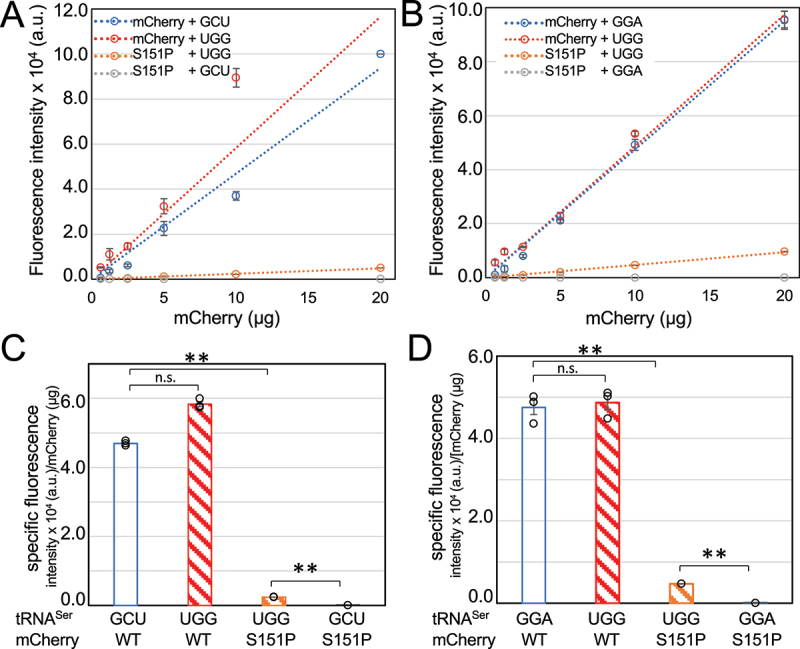
Fluorescent measurements of purified mCherry variants. mCherry proteins variants were purified and fluorescence (ex 570 nm, em 610 nm) of each serial dilution (a, b) was measured. Based on linear regression of the data (A,B dotted lines), the slope is the specific fluorescence or fluorescence intensity per amount of mCherry protein (c, d). The wild-type or Ser151Pro mCherry variants were produced with co-expression of either the (a) tRNA^Ser^_GCU_ or a UGG anticodon mutant or with the (b) tRNA^Ser^_GGA_ or a UGG anticodon mutant. According to specific fluorescence, the fluorescence recovery or serine mis-incorporation level is (c) 4.1% for the tRNA^Ser^_GCU_ derived proline decoding mutant and (d) 9.7% for tRNA^Ser^_GGA_ derived mutant. Statistical significance is based on ANOVA single factor analysis (n. s. – not significant, ** p < 0.01).

**Figure 6.**
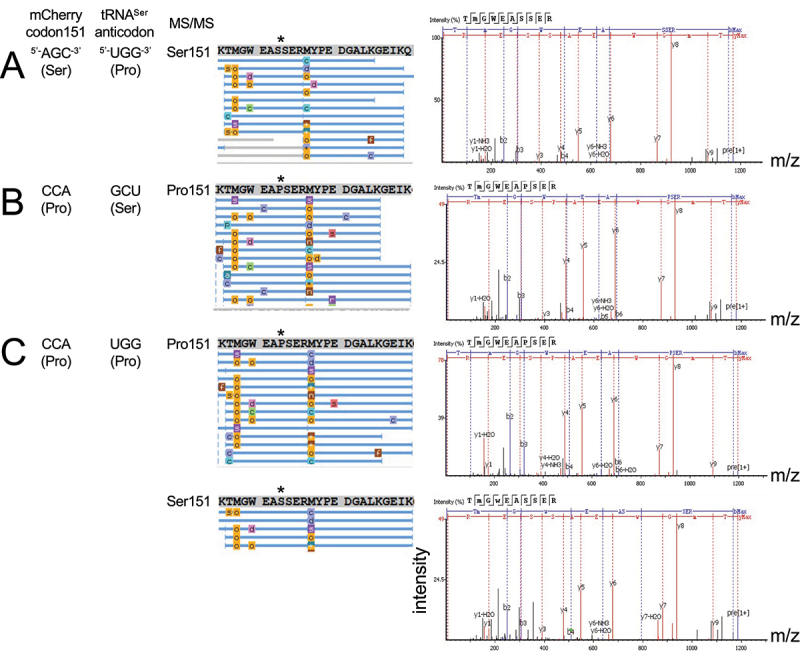
Tandem mass spectrometry analysis reveals Ser mis-incorporation in mistranslating cells at Pro151. MS/MS coverage of purified and trypsin digested mCherry variants is represented as a schematic with each blue bar representing an identified peptide. MS/MS spectra for representative peptide hits are shown (right). Cells co-expressing wild-type mCherry with a mutant tRNA^Ser^_UGG_ (a) showed only Ser151 as expected. Cells co-expressing the mutant mCherry with a wild-type tRNA^Ser^ (b) showed only Pro151. Mistranslation was readily detected in cells expressing both mutant mCherry and mutant tRNA (c) where multiple high-quality spectra were identified for peptides containing either Pro151 or Ser151.

**Figure 7.**
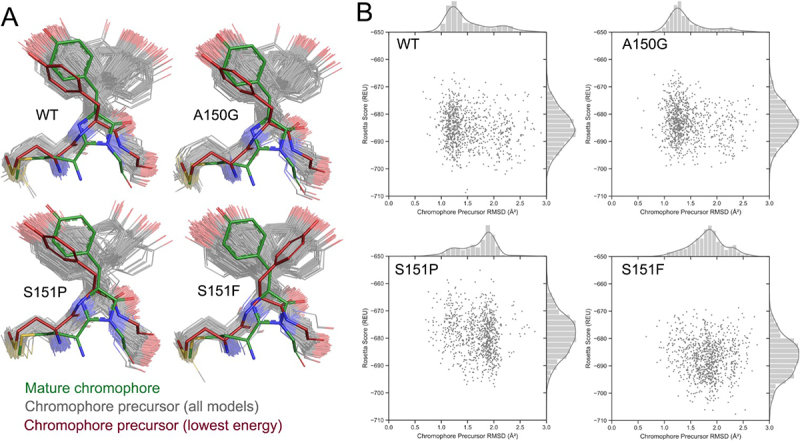
Conformational sampling explains the mechanism of fluorescence inhibition and rescue. A set of 1,000 plausible conformations of the entire mCherry precursor protein were generated in Rosetta for WT, Ala150Gly, Ser151Pro and Ser151Phe mutants. (a) The distribution in chromophore precursor position was compared to that of the mature chromophore in the 2H5Q mCherry crystal structure [31]. (b) The position distribution for each mutant is represented as RMSD values (x-axis) of the chromophore precursor atoms compared to the initial starting model. The Rosetta Score (y-axis), a metric used to estimate relative conformational stability, is also shown for each generated model.

Please make this an all figure in color. I replaced the images with color jpeg images and included the original pdf images for Figs 1-7 in the attachments.

Ths is a color figure.

### Growth of mistranslating cells

As mistranslation may cause a growth defect, we measured cell growth for each strain. To accurately quantify the cell growth in each strain, we performed microplate growth assays. The mCherry fluorescence was measured simultaneously (Figure 3). *E. coli* BL21 cells harbouring plasmids for the tRNA and the mCherry variants were grown in pre-cultures of biological replicates from three independent colonies. Cells were incubated at 37°C while shaking and monitored over a 16 hr time course. A_600_ and fluorescence intensity (ex 570 nm, em 610 nm) were recorded in 15 min intervals. Overall, as we observed in solid media (Figure 2), each cell line showed similar growth (Figure 3(a,c)). In cells expressing the tRNA^Ser^_UGG_ mutant derived from the GCT-1-1 tRNA^Ser^ gene, we only observed a slight growth defect in mistranslating cells also expressing the Ser151Pro mCherry mutant (Figure 3(a)). In both strains expressing a tRNA^Ser^_UGG_ mutant derived from the GGA-1-1 tRNA^Ser^ gene, we observed a slight growth defect compared to cells expressing wild-type tRNA^Ser^ (Figure 3(c)).

This is a color figure

### mCherry reporter illuminates mistranslation events in E. coli cells

In the same microplate assay, we monitored fluorescence of mCherry produced in each well. Both normal and mistranslating cells expressing wild-type mCherry showed strong fluorescence intensity that increased continually and saturated the detector just before the end of the time course (Figure 3(b,d)). We observed no fluorescence intensity above the background for mCherry Ser151Pro in normal cells expressing wild-type tRNA. In the mistranslating cells expressing either of the tRNA^Ser^_UGG_ mutants, significant fluorescent recovery was recorded (Figure 3(b,d)). In mistranslating cells with a tRNA^Ser^_UGG_ derived from the GCT-1-1 gene, the mCherry mutant displayed fluorescence at the 4-hour mark that increased until the end of the 16 h time course (Figure 3(b)). Mistranslating cells expressing a tRNA^Ser^_UGG_ derived from the GGA-1-1 gene showed increasing rescued fluorescence at the 4-hour mark until the 12-hour time point before plateauing (Figure 3(d)). The results confirm that the reporter quantifies tRNA-dependent mistranslation in living cells.

We then used these data to calculate the maximal level of mis-made protein and the rate of mistranslation induced by each tRNA^Ser^ variant (Table 1). By comparing the maximal fluorescence per cell reached during the time course (Figure 3), we observed a significant rescue of fluorescence in the mistranslating cells (Figure 4). The maximal level of fluorescence reports how much mis-made mCherry corresponds to mistaken incorporation of Ser at the Pro151 codon. To estimate the fluorescence per cell, we normalized the fluorescent data by the A_600_ value at the same time point. These data suggest that tRNA^Ser^_GCU_ derived UGG anticodon mutant led to a maximal level of 12% mis-made mCherry protein, while the level we recoded for cells expressing the GGA-1-1 derived tRNA^Ser^_UGG_ represented a maximal level of 15% serine mis-incorporation at the Pro151 codon. In terms of mistranslation rates over time, we determined the line of best fit to the linear portion of the mCherry production curve (Figure 3(b,d)). For tRNA^Ser^_GCU_ and tRNA^Ser^_GGA_ derived proline-decoding mutants, the rate of mistranslation in the mCherry mutant was ~22% of the rate we observed for production of the wild-type mCherry protein. We observed no significant difference in either the maximal level or rate of fluorescence production for wild-type mCherry produced in normal compared to mistranslating cells (Figure 4). Because the mis-made protein production appears to lag until the 4-hour mark in the time courses (Figure 3(b,d) insets), these somewhat high rates of mistranslation occurred for shorter times than wild-type mCherry protein production, thus amounting to the somewhat lower maximal levels of mis-made protein (~12-15%) we observed per cell (Figure 4(a,b)).

**Table 1.** Ser mis-incorporation levels in cells and purified mCherry proteins

mCherry variant	tRNA^Ser^ anticodon	Maximal fluorescence per cell	Relative rate of fluorescence per cell	Relative specific fluorescence of purified mCherry	MS/MS Peptide Count # S, P151
WT	GCT	91 ± 11%	78 ± 23%	80.0 ± 0.5%	-
WT	UGG	100 ± 30%	100 ± 32%	100 ± 1%	-
Ser151Pro	GCT	0.00 ± 0.03%	0.01 ± 0.01%	0.010 ± 0.003%	-
Ser151Pro	UGG	12 ± 2%	21.0 ± 1.5%	4.1 ± 0.2%	S 5 P 17
		**Maximal level of error per cell/codon:**	**Relative rate of error per cell/codon:**	**Level of error/codon in soluble fraction:**	
***Error levels*** ***at CCA151***		**12 ± 2%**	**21.0 ± 1.5%**	**4.1 ± 0.2%**	29%
WT	GGA	72 ± 20%	67 ± 13%	98 ± 3.5%	-
WT	UGG	100 ± 9%	100 ± 9%	100 ± 3.5%	-
Ser151Pro	GGA	0.00 ± 0.01%	0.00 ± 0.05%	0.005 ± 0.003%	-
Ser151Pro	UGG	15.0 ± 0.5%	23 ± 5%	9.7 ± 0.1%	S 9 P 19
		**Maximal level of error per cell/codon:**	**Relative rate of error per cell/codon:**	**Level of error/codon in soluble fraction:**	
***Error levels*** ***at CCA151***		**15.0 ± 0.5%**	**23 ± 5%**	**9.7 ± 0.1%**	47%

We quantitated translation error in 3 ways: 1) We calculated the maximal error level per codon per cell (column 3) by dividing the maximal fluorescence intensity at the end of the time course (Figure 3) by the number of cells recorded at that time using the standard conversion (A_600_ unit = 8 × 10^8^ cells/mL); 2) We quantified the rate over time at which the mis-made protein accumulated per CCA151 codon in cells (column 4) by determining the slope of the linear phase of mCherry fluorescence production (between the 7 and 12 hour time points, Figure 3) to determine the rate of fluorescence increase over time for each strain; and 3) we determined the level of error in the purified mCherry reporters from large preparative cultures, which represents the total error level per CCA151 codon in the soluble protein fraction. The values (column 5) are based on the specific fluorescence measurement of pure mCherry variants (Figure 5). The data were normalized relative to the wild-type mCherry produced in mistranslating cells, which was set to 100%. We found no significant difference between the levels of mCherry produced in normal versus mistranslating cells (Figures 4(c,d) and 5(c, d)). Finally, we included a summary of spectral counts of peptides identified by MS/MS (Figure 6 and Table S3) as containing Ser151 or Pro151 in response to the CCA151 codon in mistranslating cells.

This is a color figure

This is a color figure

This is a color figure

### Purified mCherry confirms fluorescence recovery from mistranslation

To assess the level of mistranslation in the soluble protein fraction, we purified mCherry variants and characterized their specific fluorescence. The His-tagged wild-type and Ser151Pro mCherry variants were purified via affinity chromatography from cells expressing wild-type or mistranslating tRNAs (Figure S3). The purified variants were balanced to the same concentration and fluorescence (ex 570 nm, em 610 nm) was measured across a dilution series for each mCherry variant (Figure 5).

In a plot of fluorescence as a function of mCherry concentration, we determined the specific fluorescence or the fluorescence intensity per amount of mCherry protein for each variant. We first compared the fluorescence of wild-type mCherry produced in either normal or mistranslating cells. We found no significant difference in the specific fluorescence of wild-type mCherry purified from normal cells expressing wild-type tRNAs compared to mistranslating cells expressing either of the Pro-decoding tRNA^Ser^_UGG_ mutants (Figure 5(c,d), Table S2). The data suggest that serine mis-incorporation at other proline codons (Table S3) in mCherry has minimal impact on the protein’s red fluorescent properties.

*E. coli* produces significant levels of each mCherry variant despite mistranslation. We recorded the yield of wild-type or Ser151Pro mCherry produced in each cell line (Figure S3E). We Western blotted for the His-tagged mCherry from lysate representing the same number of wild-type cells producing wild-type, Ser151Pro, or Ser151Phe mCherry. All variants were produced to a similar level (Figure S2B). We observed more variability in yield from large preparative cultures. We did not, however, record significantly more production of either mCherry variant in comparable normal cells versus mistranslating cells, nor was there a statistically significant difference in the yield of Pro151 versus Ser151 mCherry in cells expressing the same tRNA or its derivative variant (Figure S3E). We did find mCherry Pro151 was produced with somewhat reduced yield (p = 0.044) in cells expressing the tRNA^Ser^_GGA_ derived UGG mutant compared to cells co-expressing wild-type mCherry with the other wild-type tRNA^Ser^_GCU_.

By comparing the specific fluorescence of each variant, we found that the purified mCherry Ser151Pro recovered 4.1% of the wild type mCherry-specific fluorescence when co-expressed with the tRNA^Ser^_UGG_ derived from the GCT-1-1 tRNA^Ser^ gene. Thus, the purified mCherry Ser151Pro contains 4% fluorescent protein, indicating a 4% level of serine mis-incorporation at Pro151 in the soluble fraction. The tRNA^Ser^_UGG_ mutant derived from GGA-1-1 tRNA^Ser^ gene enabled fluorescent recovery of 9.7% in the purified mutant relative to wild-type mCherry. The data demonstrate that different levels of mistranslation result from different tRNA^Ser^ gene variants. *E. coli* cells are known to tolerate rates of mistranslation of up to 10% without significant impact on cell growth [14] as we observed here.

### Mass spectrometry confirms mistranslation in mCherry

Tandem mass spectrometry (LC-MS/MS) analysis confirmed mis-incorporation of Ser at the Pro151 codon in cells expressing the mutant tRNAs (Figure 6 and S4). To validate serine mis-incorporation at proline codons, we conducted MS/MS analysis of tryptic peptides derived from each of the purified mCherry variants. We conducted the analysis on wild-type and mutant mCherry proteins produced in cells expressing wild-type tRNA^Ser^_GCU_ (Figure 6) or tRNA^Ser^_GGA_ (Figure S4) or the corresponding UGG anticodon mutant tRNAs. We found evidence of only Ser151 in protein from cells expressing the wild-type mCherry (Figure 6(a) and S4A). In cells expressing the mutant mCherry Ser151Pro and a wild-type tRNA, only peptides corresponding to Pro151 were identified (Figure 6(b) and S4B). Finally, cells expressing the mutant tRNA and mutant mCherry show both accurate translation of Pro151 and multiple, high-quality peptides hits demonstrating mis-incorporation of Ser at the Pro (CCA) codon at position 151 (Figure 6(c) and S4C). The data confirm that fluorescence rescue results from Ser mis-incorporation at the Pro151 codon.

We also searched the MS/MS spectra and identified Ser mis-incorporation at 10 of the total 12 Pro codons in mCherry Ser151Pro (Figure S5). In mCherry Ser151Pro purified from mistranslating cells, we identified 29 peptides in total representing Ser mis-incorporation and 101 peptides representing Pro incorporation at Pro codons. In wild-type mCherry produced in mistranslating cells, we found a total of 10 peptides indicating Ser mis-incorporation at the native Pro codons and 43 peptides representing Pro (Table S3). In a total analysis of mCherry purified from mistranslating cells, we identified mis-incorporation of Ser at the 1 CCA (14 peptides), 11 CCC (22 peptides) and 1 CCT (3 peptides) Pro codons included in the mCherry sequence (Table S3). The construct did not contain the CCG Pro codon. In protein purified from cells expressing the tRNA^Ser^_GCU_ derived UGG anticodon mutant, we found strong evidence of mis-incorporation at Pro90, 151, 161, and 213. In the protein from cells expressing tRNA^Ser^_GGA_ derived proline-decoder, we found Ser at some of these and additional sites, including Pro90, 103, 107, 115, 151, 157, 159, 179, 213, and 217. Although we searched the data specifically for spectra that would indicate multiple mis-incorporation events in two or more Pro codons in the same peptide, we found no peptides to support this possibility. If multiple mis-incorporations do occur, their abundance may be below the detection limit for our experiment. Finally, we searched the spectra for evidence of mis-incorporation all possible single amino acid substitutions, but we found no evidence of any other kind of mistranslation.

### Molecular mechanism of fluorescence restoration in mCherry Pro151

We employed a Rosetta-based [33] computational pipeline to investigate the structural mechanism of mCherry mutant dysfunction. The presence of non-canonical amino acids like the mature chromophore in mCherry complicates many Rosetta applications due to the empirical nature of Rosetta protocols. To circumvent this limitation, we studied the effect of mutations on chromophore maturation indirectly by sampling the conformational distribution of the mCherry precursor. First, wild-type mCherry was remodelled to harbour the chromophore precursor peptide, Met71-Tyr72-Gly73 in place of the mature CH6 chromophore. Despite the large amount of conformational freedom that was permitted in the simulation, the lowest-energy model closely resembled the crystal structure of mature mCherry with an RMSD of 2.5 Å (Figure S6). From the lowest-energy mCherry precursor structure, we generated 1,000 energy-minimized models of wild-type, Ala150Gly, Ser151Pro and Ser151Phe mCherry variants using a global structure relaxation algorithm. Each model represents a possible conformation of the corresponding mCherry variant.

Analysis of the position of the chromophore precursor within the conformer set revealed that the Ser151Pro and Ser151Phe mutations both significantly alter the conformation distribution of the Met71-Tyr72-Gly73 precursor compared to wild type. In contrast, Ala150Gly mCherry, an experimentally validated functional mutant (Figure S2), yielded a nearly identical conformation distribution as wild type (Figure 7(a)). To quantify the differences observed between the structure distributions, the RMSD of the chromophore tripeptide was calculated to the initial starting model for each of the 1,000 output models (Figure 7(b)). The distribution in RMSD values was compared between all pairs of mCherry mutants using the two-sample Kolmogorov–Smirnov (KS) test (Table 2). The KS test indicated that the differences in the chromophore position distribution of Ala150Gly and wild-type mCherry are not statistically significant. Meanwhile, Ser151Pro and Ser151Phe both have statistically significant distributions in chromophore position compared to wild-type mCherry and each other. The analysis shows that increased confirmational flexibility in the non-fluorescent Pro151 and Phe151 mutants inhibits proper formation of the mCherry chromophore.

**Table 2.** Two-sample KS test results from comparing RMSD distributions of mCherry variants

Variant 1	Variant 2	D-statistic	P-value	Interpretation*
WT	A150G	0.0895	8.96 × 10^−4^	Equivalent
WT	S151P	0.3573	8.18 × 10^−55^	Different
WT	S151F	0.4729	1.91 × 10^−97^	Different
A150G	S151P	0.4365	1.54 × 10^−82^	Different
A151G	S151F	0.5896	3.27 × 10^−133^	Different
S151P	S151F	0.1552	1.67 × 10^−10^	Different

*Interpretation of D-statistic and P-value to state whether the RMSD distributions of the two mCherry variants are equivalent or significantly different at an α-level of 10^−6^ (D statistic critical value = 0.0933).

This is a color figure

## Discussion

### Mistranslation reporters for living cells

Here, we developed a novel mCherry reporter for serine mis-incorporation that can quantify protein synthesis error rates per codon and levels of mis-made protein in living bacterial cells. We found that both Pro151 and Phe151 variants eliminated fluorescence, and our studies demonstrated that two different proline decoding tRNA^Ser^ gene mutants rescued specific mCherry Ser151Pro fluorescence to differing levels. In future work, we will assess the ability of other Ser151 mutants to detect serine mis-incorporation at different codons. We anticipate this mCherry reporter should be directly portable to mammalian cells or other model systems where mCherry is routinely used. For example, the reporter can be used to assess mistranslation resulting from naturally occurring human tRNA variants [1] in a physiologically relevant context [22]. Other areas of application include studies on microbial pathogens. Ser mis-incorporation can be intentional and contribute to disease from the model human pathogen *Candida albicans* that tolerates extreme (97%) serine mis-incorporation at leucine CUG codons [36]. Indeed, the yeast evolved to require ambiguous decoding, which can help enhance growth rate in response to environmental stress [37].

Our approach was inspired by reporters that were designed for sensitivity to other types of amino acid mis-incorporation. Many of these studies focused on mutants of aminoacyl-tRNA synthetases that cause mis-aminoacylation of different native tRNA species. One of the first mistranslation sensitive reporters was designed using enhanced GFP (eGFP) to detect threonine mis-incorporation at valine codons [38]. A valine mutation to GFP residue Thr65, which is a component of the chromophore, inactivated green fluorescence. In NIH 3T3 mouse fibroblast cells, a transgenic editing defective valyl-tRNA synthetase allele (T535P) was expressed to induce threonine mis-incorporation at valine codons. The normal ValRS possesses an editing activity that cleaves mis-charged threonine from tRNA^Val^, thus preventing the mis-aminoacylated tRNA substrates from reaching the ribosome. The editing defective ValRS, however, produces both Val-tRNA^Val^ and Thr-tRNA^Val^. Co-expression of the eGFP Thr65Val reporter with the editing defective ValRS led to a significant restoration of fluorescence compared to cells with wild-type ValRS. The authors report a 15% increase in fluorescence of the mutant reporter in mistranslating cells, which showed increased apoptosis and decreased cell growth. Because the study did not measure the fluorescence from wild-type GFP in the same cells, a mistranslation rate or level was not reported based on fluorescence. Label-free mass spectrometry and spectral counting was used to estimate the mistranslation rate of 10–18% per valine codon. The work clearly showed that threonine mis-incorporation at valine codons revived the fluorescent activity of the GFP by effectively reverting the mutation in a portion of the protein produced.

We previously engineered GFP Asp129Pro to detect alanine incorporation at proline codons in yeast [15] and mammalian cells [16] resulting from an alanine accepting tRNA^Pro^ mutant. In this example, Ala129 restored fluorescence similarly to the native Asp129 residue. We did find, however, a Pro129 mutant was not suitable to detect serine mis-incorporation with GFP. Luciferase and dual-luciferase constructs have also been used to measure either glutamine misincorporation at glutamate codons or asparagine misincorporation at aspartate codons [39].

Fan *et al*. used super-folder (sfGFP, Thr203Ser/Ala) to generate a reporter for mis-incorporation at Thr codons as a result of fluorescent dimming [40]. The authors generated a GFP variant with only a single Thr codon at position 203 by replacing all other native Thr residues with Ser or Ala. Mistranslation was induced by expressing an editing defective threonyl-tRNA synthetase (ThrRS) that ligates Thr and also Ser and Ala to tRNA^Thr^. Lack of the natural editing activity means that the Ser- and Ala-tRNA^Thr^ species produced by the ThrRS catalytic site will not be hydrolysed and will then be used in translation. The engineered sfGFP reporter was based on loss of fluorescence to report mis-incorporation. Correct translation of Thr codons led to active sfGFP, while mis-incorporation of Ser or Ala at the same site eliminated fluorescence in proportion to the translation mistake rate. Based on this reporter, the editing defect ThrRS (C182A) resulted in ~3% mis-incorporation rate [40], which was similar to that observed using a quantitative mass spectrometry approach relying on standard peptides [41].

Regarding serine mis-incorporation at proline codons that we studied, we found no significant difference between mCherry produced in wild-type or mistranslating cells, suggesting that eliminating native proline codons may not be necessary and can be accounted for with appropriate controls. Despite all these studies and advances, there are still no reporters for most types of amino acid mis-incorporation. Thus, additional tools will be needed to investigate the complete palette of mistranslation events in model organisms.

### Estimating the rate of mistranslation

Determining the rate of errors in protein synthesis remains a challenge, especially in applications for live cells. We highlighted above and added to the catalogue of fluorescent proteins used detect and indeed quantify the rate and level of mistranslation in cells and in purified proteins produced from cells. Although proline codons at other locations may experience different levels of error depending on codon context, our reporter provides a standardized and quantitative live-cell approach to measure the impact of different tRNA mutants or other gene variants that compromise translation fidelity in cells.

We recorded somewhat different levels of fluorescence recovery in cells in the microplate versus in protein purified from larger preparative cultures. In comparing the two tRNA^Ser^_UGG_ variants, we observed 15% fluorescence recovery the mutant derived from tRNA^Ser^_GGA_ and a reduced level of mis-incorporation (12%) for the tRNA^Ser^_GCU_ mutant in cells in the microplate. In the microplate assay, we noted a lag before fluorescence recovery of the Ser151Pro mutant could be observed. At early time points, the level of mistranslated protein is too low to be detected above the background. Only when sufficient mistranslated protein accumulates, can we observe the restored mCherry fluorescence above the background.

In the proteins we purified from larger cultures, we found 10% mis-incorporation resulting from the tRNA^Ser^_GGA_ derived proline decoder and similar decreased level of 4% mis-incorporation resulting from the tRNA^Ser^_GCU_ derived mutant. The mistranslation rate for purified protein is somewhat lower as the purification process only captures soluble proteins. The protein purification removes all insoluble protein and protein aggregates, which does include a proportion of the mCherry. Thus, with the purified mCherry, we measured the mistranslation rate per codon in the soluble protein fraction. Both cell-based measurements and data from purified protein agree that the tRNA^Ser^_GGA_ derived mutant is more effective at serine mis-incorporation. Cells expressing the tRNA^Ser^_GGA_ derived mutant also showed a slight but consistent growth defect (Figure 3(c)), whereas no significant growth defect was observed in cells expressing the less effective tRNA^Ser^_GCU_ derived mistranslator.

In addition to fluorescent or luminescent reporter systems, several studies have used mass spectrometry to estimate the level of mistranslation in bacterial cells [41,42] and other model systems, including yeast [43] and mammalian cells [38]. Many of these semi-quantitative approaches rely on spectral counting [42]. The error rate per codon is estimated by counting the number of spectra for peptide ions [43] identified as mis-incorporated divided by the number of peptides representing proper incorporation across the proteome or in individual proteins. Based on this approach, we estimated the level of mistranslation by examining spectral counts. Spectral counting led to an over-estimation of the level of Ser mis-incorporation at position 151 by a factor of 5 to 7 (Table S3). For the tRNA^Ser^_GCU_ derived mutant, we recorded 4.1 ± 0.2% mis-incorporation according to fluorescence data, while the same protein showed 9 peptide ions assignable to mis-incorporation of serine and 19 to Pro151. The tRNA^Ser^_GGA_ derived mutant gave rise to 9.7 ± 0.1% mis-incorporation level in purified mCherry, and the same protein analysed by MS/MS showed 5 spectra assignable to mis-incorporation and 17 assignable to accurate translation. We also counted spectra at all of the Pro codons in mCherry (Figure S5), providing a global mis-incorporation level based on spectral counts across the mCherry protein (Table S3) of 17 ± 13% for the stronger mis-translator and 34 ± 5% for the weaker mistranslator derived from tRNA^Ser^_GCU_. Spectral counting across different sites was highly variable and over-estimated the 10% and 4% mis-incorporation levels we measured at Pro151 according to fluorescence. The data suggest spectral counting does not have sufficient resolving power to accurately measure mis-incorporation levels. In summary, we found the MS/MS data essential for identifying mis-incorporation events at multiple Pro codons; however, the use of semi-quantitative approaches based on these data did not accurately estimate mis-incorporation levels. Overall, analysis of the error levels per codon suggests that tRNA^Ser^_GGA_ mistranslates at a higher frequency than tRNA^Ser^_GCU._ The tRNA^Ser^_GGA_ may be expressed at a higher level than the GCU isoacceptor, or the GGA-derived mutant may decode proline codons more efficiently on the ribosome.

Recent studies have used quantitative mass spectrometry approaches to assess mis-incorporation in individual proteins [41] or across the proteome, including methods for relative quantitation using stable isotopic labelling (SILAC) [42] or absolute quantitation using synthetic amino acid standards [44]. The *E. coli* leucyl-tRNA synthetase (LeuRS) editing defective mutant (D345A) allows significant production of tRNA^Leu^ aminoacylated with the non-standard amino acid nor-valine (Nva) [45]. The result was mis-incorporation of nor-valine at leucine codons. SILAC was used to generate light-labelled normal cells (Lys-0) and heavy-labelled (Lys-4) mistranslating cells expressing the LeuRS mutant [42]. The degree of mis-incorporation was quantified similarly to approaches used to quantify protein modification [46]. Based on relative quantitation of light and heavy peak intensity ratios and using data based on 350 different Leu sites, the authors found 16% median occupancy of Leu sites with Nva mis-incorporated. The high rate based on SILAC contrasts with a rate of 1.3% mis-incorporation that the same authors calculated based on spectral counting. They found mis-incorporation of Nva in cells with either wild-type LeuRS (0.15% 40 Nva/26414 Leu peptides) or the editing defective mutant (1.3% 409 Nva/30646 Leu peptides). A different study used total amino acid analysis and synthetic amino acid standards to quantify the global level of mis-incorporation of a non-standard amino acid (meta-Tyr) at phenylalanine codons [44]. Together these studies suggest that further development is required to use mass spectrometry data to provide accurate quantitation of mistranslation levels in whole proteomes or in individual proteins.

SILAC approaches can provide relative quantitation on the same peptide in two different conditions or cell lines, but, as the above example illustrates, SILAC cannot directly compare a properly translated peptide to a mis-translated peptide in the same cell, nor can it be used for relative quantitation of different peptides in different cells. In our mCherry reporter, SILAC could be used to quantify the relative level of Ser151 or Pro151 in normal versus mistranslating cells, but since the level of mistranslation in normal cells is low and likely below the detection limit, the relative quantitation of mis-incorporation between normal and mistranslating cells would be highly variable. This situation reveals directions for future work and underscores the need for additional fluorescent or other reporters to quantify amino acid mis-incorporation in live cells and proteins.

### Computational analysis and design of mistranslation reporters

Direct simulations of chromophore maturation in fluorescent proteins is computationally expensive, as systems involving bond formations typically require computation on the quantum-mechanics/molecular-mechanics level (QM/MM) to be simulated reliably [47]. To circumvent the need for QM/MM computations, we studied the effect of mutations on chromophore maturation indirectly by sampling the conformational distribution of the mCherry precursor. Since the mCherry precursor structure contains only canonical amino acid residues, we can employ fast empirical computation methods that have been refined specifically for protein structure modelling. Using Rosetta, it is feasible to generate a large number of potential conformers of the mCherry fluorescent precursor structure. From these conformation distributions, we determined that Ser151Pro and Ser151Phe mutant dysfunction appears to be a result of a defect in chromophore maturation due to altered conformational freedom at the chromophore precursor peptide.

We showed that differences in the distribution of conformer sets can be detected using the Kolmogorov–Smirnov test. Together, our mCherry modelling methods with KS test metrics form the basis of a feasible and rapid computational approach to predict the functional status of novel mCherry variants. Using a set size of 1,000 conformers, the functional status of an mCherry variant can be predicted in under 1 hour using only 480 cores on a cloud computing cluster. The conformer set size may be decreased at the cost of KS test sensitivity to allow for high-throughput searches of mCherry or other fluorescent protein variants. Such high-throughput methods can be used to computationally design reporters for other mistranslation systems that may be otherwise challenging to design rationally. In future, we envision that the pipeline can be adapted to search for fluorescent reporters for other important cellular events, including to probe the dynamic regulation of protein modifications, such as phosphorylation or acetylation.

## Conclusion

We described a novel reporter for serine mis-incorporation based on fluorescence rescue in mCherry. We used the system to measure error rates and levels in live cells that mis-incorporated serine at proline codons as a result of two different mutant tRNA^Ser^ genes. We characterized the amount of mis-made protein in cells, and following purification of the reporter, we were able to measure the level of mis-incorporation in the soluble protein fraction associated with each tRNA mutant. Mistranslation was confirmed by mass spectrometry. We also used statistical conformational sampling simulations to show that the Ser151Pro mutation in mCherry shifts the conformational distribution of the chromophore precursor and thereby disrupts chromophore maturation, resulting in a fluorescently dead mCherry protein. Despite mis-incorporation levels of 10–15% per proline codon, we measured only slight growth defects in *E. coli* cells. The data confirm the robustness of bacterial cells to errors in protein synthesis.

## Supplementary Material

Supplemental Material
