# Supplementary material for: A novel fluorescent reporter sensitive to serine mis-incorporation
Source: RNA Biol. 2022 Feb 15;19(1):221–33. doi: 10.1080/15476286.2021.2015173 (PMC8855846; doi:10.1080/15476286.2021.2015173)
Supplement: Supplemental Material [file KRNB_A_2015173_SM2889.zip › supplementary/downloadFromZipFile.pdf]

Supplemental material for:

A novel fluorescent reporter sensitive to serine mis-incorporation

Peter Rozik[1], Robert Szabla[1,**], Jeremy Lant[1,**], Rashmi Kiri[1], David E. Wright[1], Murray Junop[1], & Patrick O'Donoghue[1,2,*]

[1]Department of Biochemistry, [2]Department of Chemistry, The University of Western Ontario, London, Ontario N6A 5C1, Canada.

*Correspondence: patrick.odonoghue@uwo.ca
**These authors contributed equally.

Supplemental Methods

Western blot analysis. Lysates were separated on standard sodium dodecyl-sulphate polyacrylamide gel electrophoresis (SDS-PAGE) (15% acrylamide) and transferred to polyvinylidene difluoride (PVDF) membranes using a Trans-Blot Turbo Transfer System (BioRad, Hercules, CA, USA). Membranes were incubated for 1 hour in blocking solution (3% bovine serum albumin (BSA), 0.1% Tween 20, 1% phosphate buffered saline (PBS)) before adding α-His antibody (MA1-21315, ThermoFisher Scientific) in 3% BSA, 1 × PBS-T (1:1000). Membranes were incubated with primary antibody overnight at 4 °C and washed for 3 × 10 minutes in washing solution (1% BSA, 0.1% Tween 20, 1% PBS) at room temperature, incubated with fluorescence IRDye 700CW goat anti-mouse IgG (LI-COR) in 1% BSA, 1 × PBS-T (1:10000) for 2 hours at room temperature, washed 3 x 10 minutes in 1 x PBS-T, and 10 minutes in 1 × PBS. The membrane was imaged on the Odyssey Classic (LI-COR).

Initial modeling of mCherry precursor structure. First, 3-mer and 9-mer Rosetta fragment files were generated for the WT mCherry precursor sequence using the Robetta fragment server (https://robetta.bakerlab.org/fragmentsubmit.jsp). To generate the input structure for the Rosetta Kinematic Loop Closure with Fragments (KIC) protocol, the chromophore in PDB 2H5Q was removed and replaced with Met-71, Tyr-72, Gly-73 in Coot (numbered 66, 67 and 68, respectively in 2H5Q). The initial positions of residues 56-79 are ignored by Rosetta KIC in the following step, so the exact positioning of the MYG peptide does not have any effect on KIC output. A ".loops" file containing the line '`LOOP 53 76 0 0 1`' was generated to dictate which residues are to be remodeled. Finally, KIC was executed on the Compute Canada Niagara HPC cluster running Rosetta 3.10 with the following command to generate 1,000 output models:

```
mpirun --bind-to none [/path_to_rosetta_bin]/loopmodel.mpi.linuxiccrelease \
      -database [/path_to_rosetta_database/] \
      -loops:remodel perturb_kic_with_fragments \
      -loops:refine refine_kic_with_fragments \
      -loops:loop_file .[/path_to_loop_file] \
      -loops:frag_sizes 9 3 1 \
      -loops:frag_files [/path_to_3-mer_frags] [/path_to_9-mer_frags]
      -in:file:s [/path_to_modified_2h5q.pdb] \
      -in:file:fullatom \
      -ex1 \
```

```
      -ex2 \
      -extrachi_cutoff 0 \
      -overwrite \
      -out:file:silent 2h5q_remodel.o \
      -out:file:scorefile 2h5q_remodel.sc \
      -nstruct 1000
```

Conformational sampling of mCherry precursor structure. The lowest energy model from Rosetta KIC was mutated to either WT, Ala150Gly, Ser151Pro or Ser151Phe manually in Coot. These modified PDB files were used as input to Rosetta Relax to generate 1,000 energy-minimized conformers of the entire mCherry mutant protein. Rosetta Relax was executed on the Compute Canada Niagara HPC cluster running Rosetta 3.10 with the following command:

```
mpirun --bind-to none [/path_to_rosetta_bin]/relax.mpi.linuxiccrelease \
      -database [/path_to_rosetta_database/] \
      -relax:fast \
      -ex1 \
      -ex2aro \
      -use_input_sc \
      -relax:fast \
      -in:file:fullatom \
      -in:file:s [/path_to_lowest_energy_KIC_model.pdb] \
      -out:file:silent 2h5q_ WT_relaxed.o \
      -out:file:scorefile 2h5q _WT_relaxed.sc \
      -nstruct 1000
```

Supplemental References

1. Huang L, Pike D, Sleat DE, Nanda V, Lobel P. Potential pitfalls and solutions for use of fluorescent fusion proteins to study the lysosome. PLoS One 2014; 9:e88893.

Supplemental Tables

Table S1. Primers for cloning of mCherry libraries and mutagenesis.

Primer Name	Primer Sequence	Description
F-mCher-S74X	5′-GGCNNSAAGGCCTACGTGAAGCAC-3′	Forward mutagenic primer for S74X.
R-mCher-S74X	5′-GTACATGAACTGAGGGGACAG-3′	Reverse primer for position S74.
F-mCher-S151X	5′-GCCNNSTCCGAGCGGATGTACCC-3′	Forward mutagenic primer for S151X.
F-mCher-S151P	5′-GCCCCATCCGAGCGGATGTACCC-3′	Forward primer for S151P mutation.
F-mCher-S151F	5′-GCCTTTTCCGAGCGGATGTACCC-3′	Forward mutagenic primer for S151F.
R-mCher-S151	5′-CTCCCAGCCCATGGTCTTC-3′	Reverse primer for position S151.
F-mCher-A49X	5′-ACCNNSAAGCTGAAGGTGACCAAGG-3′	Forward mutagenic primer for A49X.
R-mCher-A49	5′-CTGGGTGCCCTCGTAGG-3′	Reverse primer for position A49.
F-mCher-A150G	5′-GAGGTGCTCCTCCGAGCGGATGTAC-3′	Forward primer for A150G mutation.
R-mCher-A150	5′-CCAGCCCATGGTCTTCTTC-3′	Reverse primer for A150G.

Table S2. Raw fluorescence data of purified mCherry protein variants

mCherry variant	$tRNA^{Ser}$ gene	anticodon variant	**specific fluorescence of purified mCherry** (fluorescence intensity (a.u.)/ mCherry (μg))
WT	GCT-1-1	GCU (Ser)	47000 ± 300
WT	GCT-1-1	GCU (Ser)	58000 ± 600
Ser151Pro	GCT-1-1	UGG (Pro)	2400 ± 10
Ser151Pro	GCT-1-1	UGG (Pro)	1 ± 3
WT	GGA-1-1	GGA (Ser)	47500 ± 1400
WT	GGA-1-1	GGA (Ser)	48700 ± 1400
Ser151Pro	GGA-1-1	UGG (Pro)	4700 ± 10
Ser151Pro	GGA-1-1	UGG (Pro)	4 ± 1

Specific fluorescence of mCherry variants was derived from the slope of the dilution curves (Fig 5). Error bars show the standard deviation of three biological replicates.

Table S3. MS/MS spectral counts for Ser mis-incorporated at Pro codons in mCherry.

mCherry variant	Pro site	$tRNA^{Ser}$ anticodon WT/mutant	Pro codon	MS/MS spectral count	**Estimated error rate/codon,** S/P spectral counts (%)
S151P	Ser151Pro	GGA/UGG	CCA	S 9/P 19	47.4%
S151P	Pro90	GGA/UGG	CCT	S 2/P 11	18.2%
S151P	Pro103	GGA/UGG	CCC	S 1/P 8	12.5%
S151P	Pro107	GGA/UGG	CCC	S 1/P 9	11.1%
S151P	Pro157	GGA/UGG	CCC	S 1/P 13	7.7%
S151P	Pro161	GGA/UGG	CCC	S 1/P 12	8.3%
S151P	Pro179	GGA/UGG	CCC	S 1/P 6	16.7%
S151P	Pro213	GGA/UGG	CCC	S 3/P 15	20%
S151P	Pro217	GGA/UGG	CCC	S1/P 18	5.6%
WT	Pro103	GGA/UGG	CCC	S 1/P 4	25%
WT	Pro115	GGA/UGG	CCC	S 1/P 4	25%
WT	Pro157	GGA/UGG	CCC	S 1/P 8	12.5%
WT	Pro161	GGA/UGG	CCC	S 1/P 8	12.5%
WT	Pro213	GGA/UGG	CCC	S2/P 5	40%
		Average for GGA-derived $tRNA_{UGG}$			19 ± 12%
S151P	Ser151Pro	GCU/UGG	CCA	S 5/P 17	29.4%
S151P	Pro90	GCU/UGG	CCT	S 1/P 7	14.3%
S151P	Pro161	GCU/UGG	CCC	S 2/P 4	50%
S151P	Pro213	GCU/UGG	CCC	S 1/P 12	12.5%
WT	Pro161	GCU/UGG	CCC	S 2/P 5	40%
WT	Pro213	GCU/UGG	CCC	S 2/P 9	22.2%
		Average for GCU-derived $tRNA_{UGG}$			28 ± 15%

A summary of spectral counts of peptides, i.e., number of peptides, identified by MS/MS (Fig 6, S5) as containing either Ser151 or Pro151 in response to each proline codon in wild-type or mutant mCherry produced in mistranslating cells.

Supplemental Figures

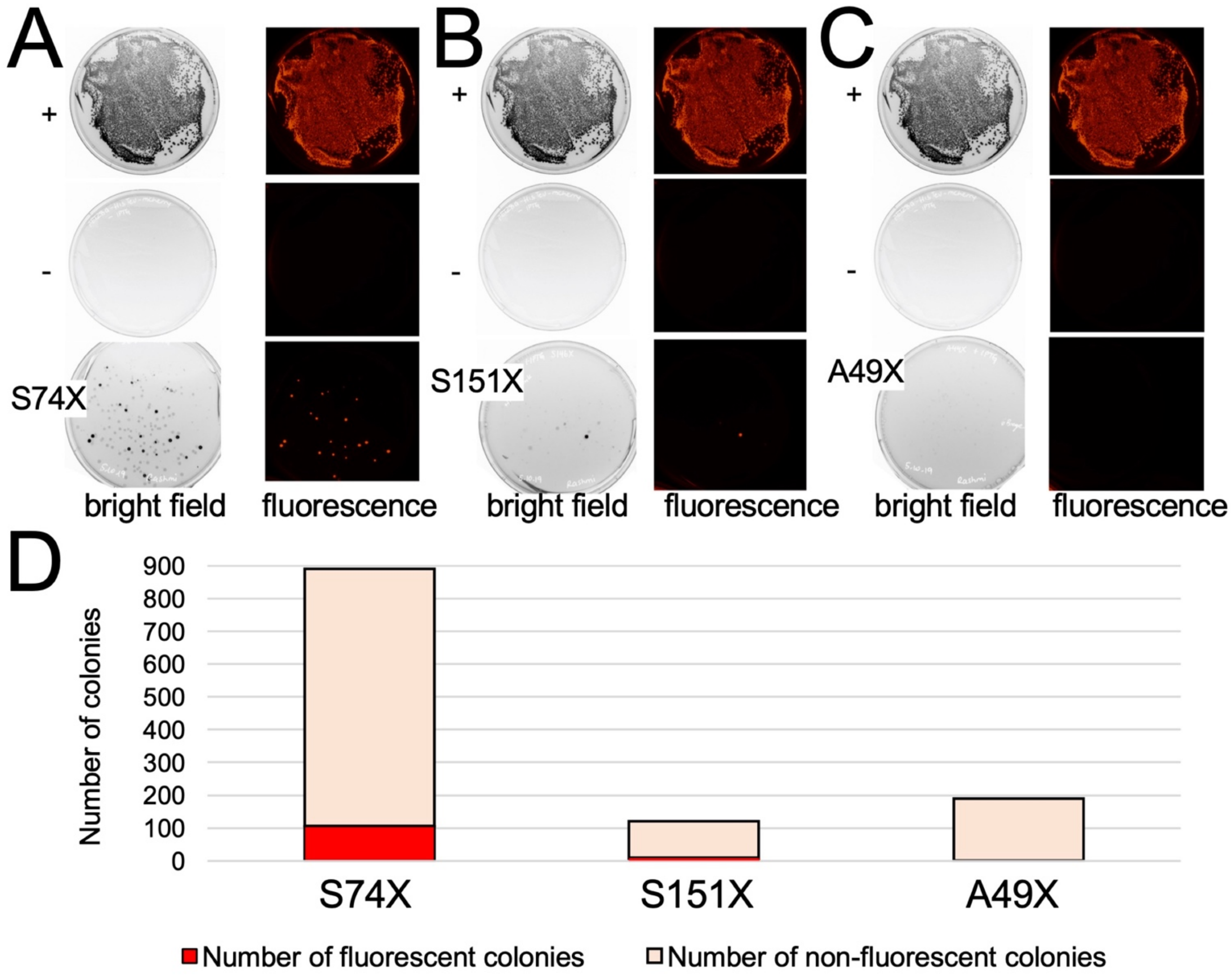

Figure S1. Analysis of mCherry mutant libraries in *E. coli*. (A) Screening of S74X, S151X and A49X mCherry libraries. *E. coli* BL21(DE3) cells contain an IPTG–inducible lac promoter–driven pET28a-His-Tev-mCherry vector. Cells containing the wildtype vector induced with IPTG are "+" while cells containing wild-type vector and no IPTG induction are "-". Cells were imaged with a ChemiDoc MP System (BioRad) under the 600/50 nm filter. (B) Total number of fluorescent and non-fluorescent colonies for each library.

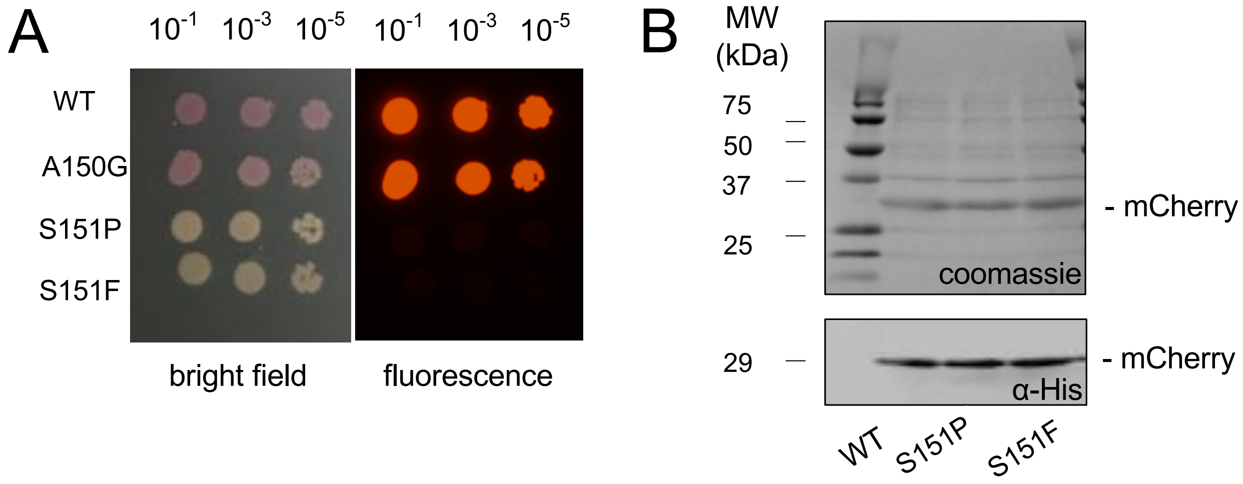

Figure S2. Growth and fluorescence of *E. coli* expressing mCherry variants. (A) *E. coli* cells expressing wild-type (pET28a-HisTev-mCherry) or mutant mCherry variants (Ala150Gly, Ser151Pro, Ser151Phe) were grown overnight in liquid media and aliquoted in serial dilutions as indicated onto an LB agar plate containing kanamycin and IPTG. Lysates representing an equivalent number of cells were loaded and visualized on a 15% SDS-PAGE gel stained with Coomassie Brilliant Blue G-250 and with Western blotting using an anti-His tag antibody (B).

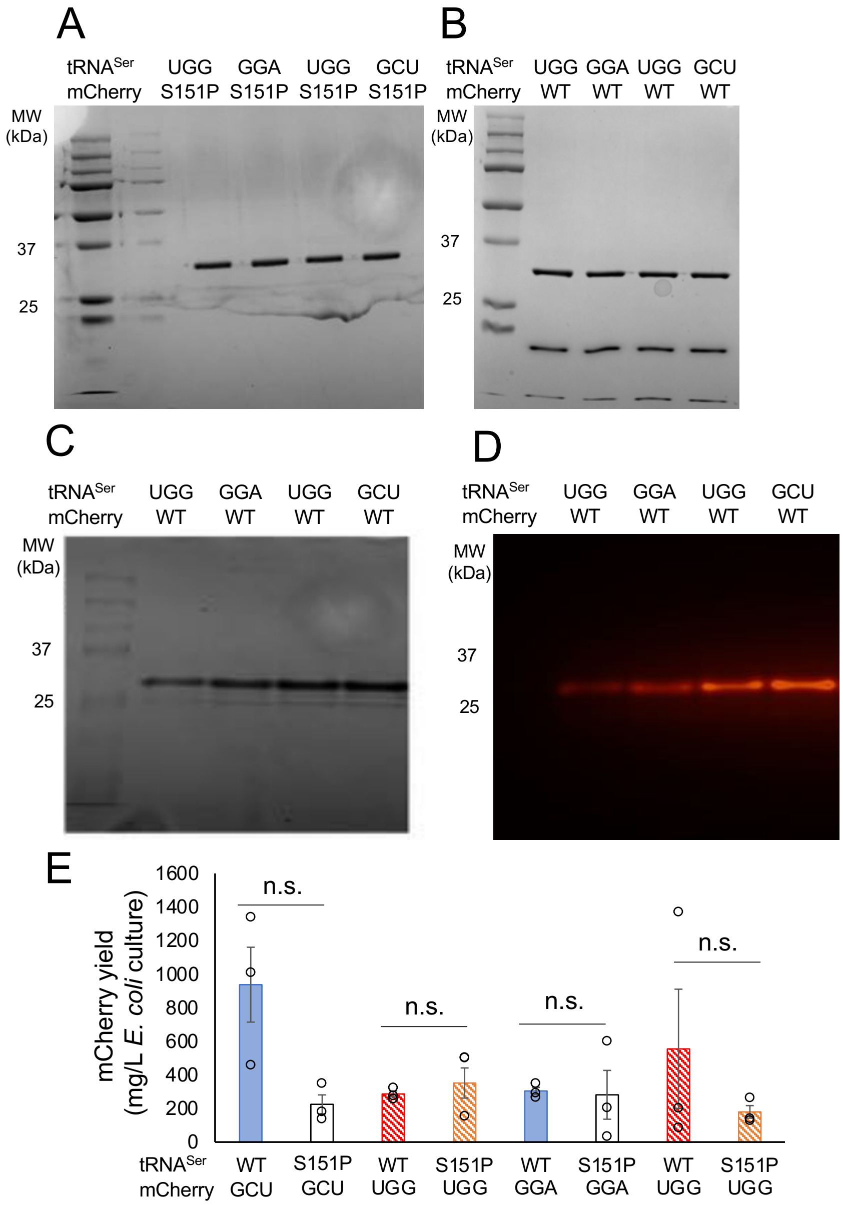

Figure S3. Purified mCherry variants. Following SDS-PAGE, gel images show affinity purified Ser151Pro (A) and wild-type mCherry (B) variants produced in cells co-expressing either a wild-type $tRNA^{Ser}$ ($tRNA^{Ser}_{GGA}$ or $tRNA^{Ser}_{GCU}$) or a derivative proline-decoding UGG anticodon variant. We observed some fragmentation of the wild-type mCherry protein (B), which was reported previously in mCherry over-production experiments[1]. (C) We found that the wild-type mCherry migrates as a single fluorescent (D) band on native PAGE (C,D), and we were unable to separate the fragments using sizing or ion-exchange chromatography. The Ser151Pro mutant lacked these fragments (A). (E) All mCherry variants were produce in high abundance (100s of mg/L *E. coli* culture). Yield for each cell line was based on three independent protein preparations. ANOVA single factor analysis indicated that none of the yields were significantly different from another (n. s. – not significant), with one exception: mCherry Pro151 was produced with somewhat reduced yield ($p = 0.044$) in cells expressing the $tRNA^{Ser}_{GGA}$ derived UGG variant compared to cells co-expressing wild-type mCherry with the other wild-type $tRNA^{Ser}_{GCU}$.

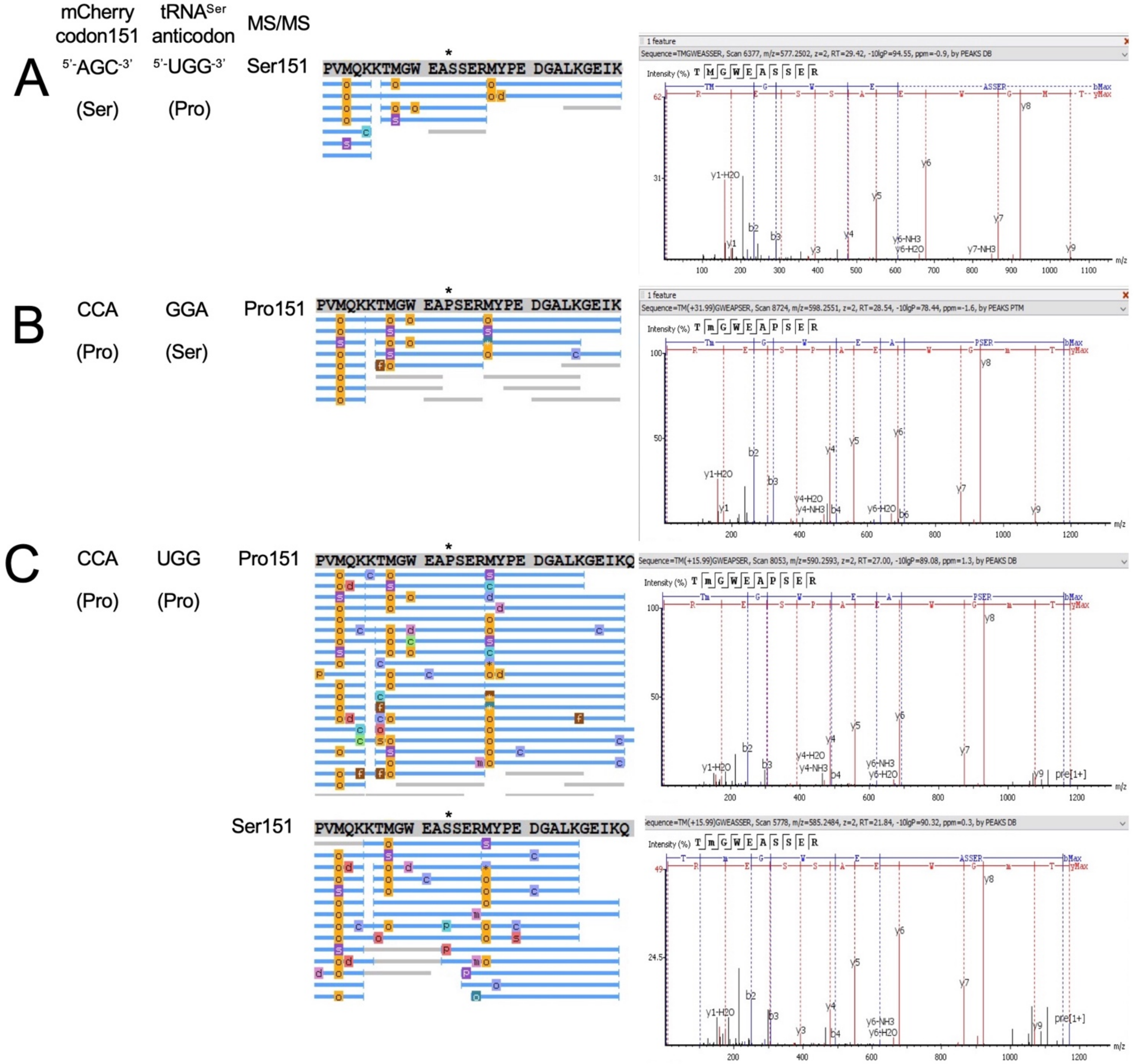

Figure S4. Tandem mass spectrometry analysis reveals Ser mis-incorporation in mistranslating cells. MS/MS coverage of purified and trypsin digested mCherry variants is shown as a schematic with each blue bar representing an identified peptide. MS/MS spectra for representative peptide hits are shown (right). Cells expressing wild-type mCherry with a $tRNA^{Ser}_{GGA}$ derived mutant $tRNA^{Ser}_{UGG}$ (A) showed only Ser151 as expected. Cells expressing the mutant mCherry with a wild type $tRNA^{Ser}_{GGA}$ (B) showed only Pro151. Mistranslation was readily detected in cells expressing both mutant mCherry and mutant tRNA (C) where multiple high-quality spectra were identified for peptides containing either Pro151 or Ser151.

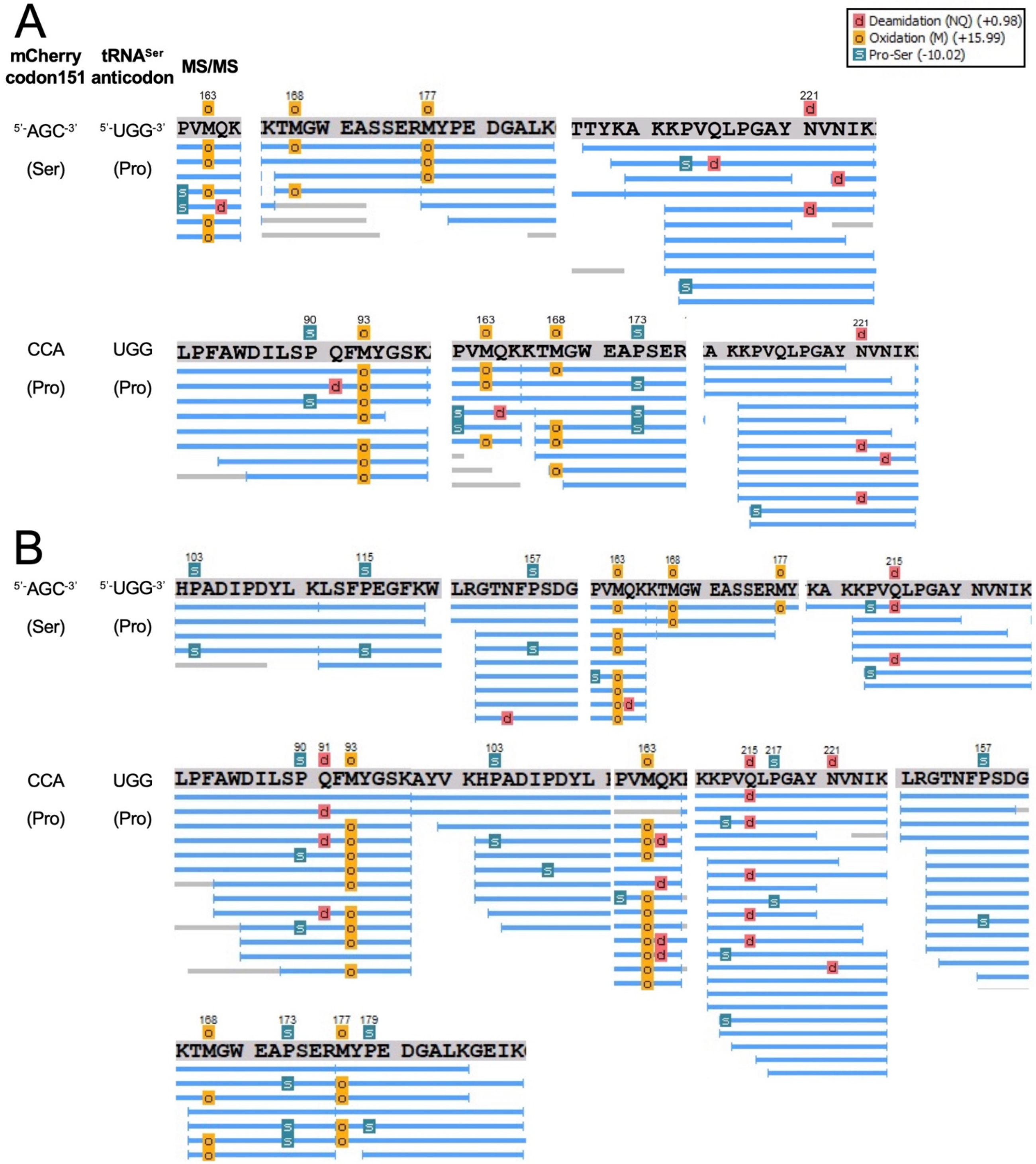

Figure S5. MS/MS coverage maps for serine mis-incorporation at native proline codons in mCherry. Coverage maps show detected peptides with serine mis-incorporation at proline codons that are marked as a "s" modification and equivalent to a loss of -10.02 Da. The MS/MS analysis identified serine mis-incorporation at native proline codons in both the wild type and mutant mCherry proteins in cells expressing either (A) $tRNA^{Ser}_{GGA}$ or (B) $tRNA^{Ser}_{GCU}$ derived proline-decoding UGG anticodon variants. We found no instances of 2 or more mis-incorporations at multiple Pro codons in a single peptide.

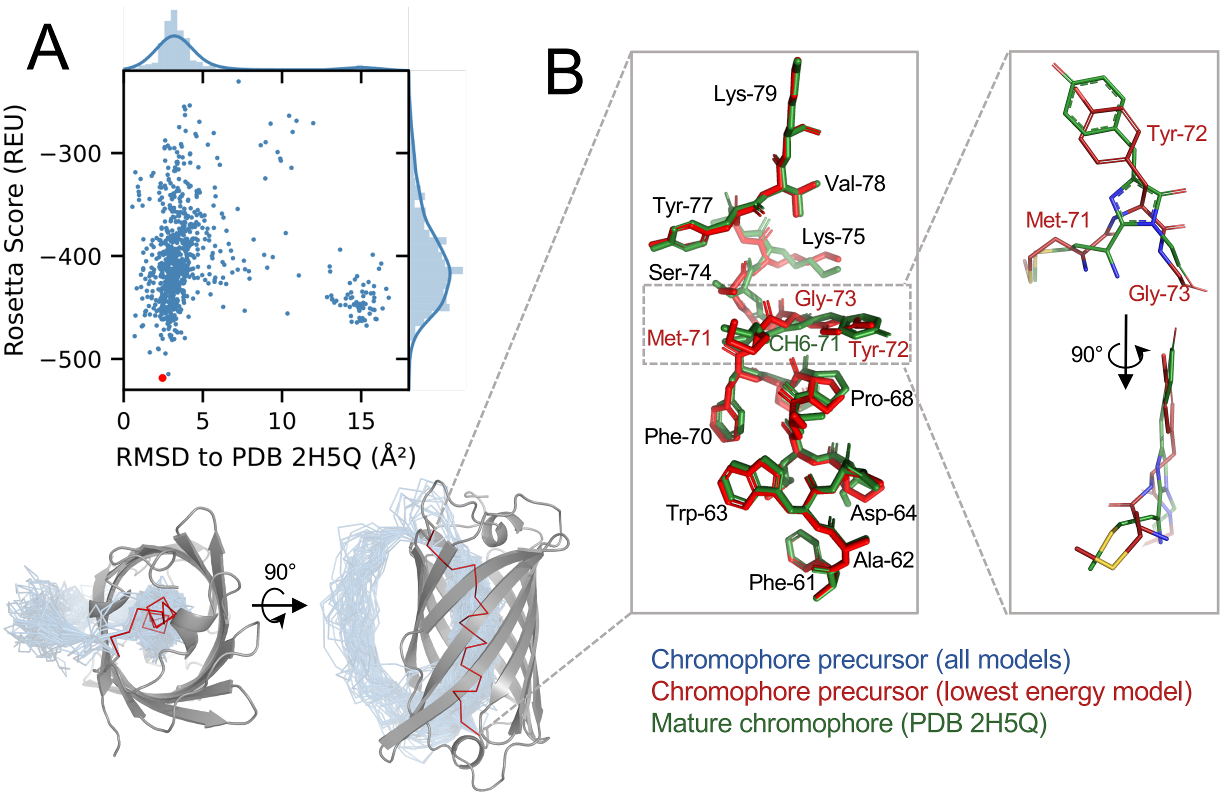

Figure S6. Modeling of mCherry chromophore precursor. Residues 56 to 79 of mCherry were remodeled from mature mCherry (PDB 2H5Q) to contain M71-Y72-G73 precursor in place of mature CH6.chromophore. (A) Funnel plot distribution of all models with the lowest energy model is highlighted (red). (B) Atomic coordinates of lowest energy model (red) compared to mature mCherry (green) and all output models (blue).
